# COUP-TFII and AKT are cancer targets pursued by SCBA award winners

**DOI:** 10.1186/2045-3701-4-57

**Published:** 2014-10-01

**Authors:** Jim Hu, Xiao-Fan Wang

**Affiliations:** Hospital for Sick Children, 686 Bay Street, Room 9.9715, Toronto, ON M5G 0A4 Canada; Duke University School of Medicine, C218 LSRC, Box 3813, Durham, NC 27710 USA

## Abstract

This thematic issue of *Cell & Bioscience* highlights review articles by Sophia Y. Tsai and Ming-Jer Tsai’s research team on roles of COUP-TFII in tumor progression and metastasis and by Hui-Kuan Lin and his colleagues on posttranslational regulation of Akt in human cancer. Drs. Sophia Tsai and Ming-Jer Tsai were the 2013 Society of Chinese Bioscientists in America (SCBA) Lifetime Achievement Award winners. Dr. Hui-Kuan Lin was the 2013 SCBA Outstanding Young Investigator Award winner.

## Editorial

At the 2013 biennial international sympoium held in Xi’an, China, the Society of Chinese Bioscientists in America (SCBA) recognized four of our finest members for their scientific achievements and future potential in biomedical research. The society awarded its Presidential Award to Dr. Xiao-Dong Wang (National Institute of Biological Sciences, Beijing, China), its Life-time Achievement Award to Drs. Sophia Tsai and Ming-Jer Tsai (Baylor College of Medicine, Houston, Texas, USA), and its Outstanding Young Investigator Award to Dr. Hui-Kuan Lin (University of Texas MD Anderson Cancer Center, Houston, Texas, USA). In this issue of *Cell & Bioscience*, we have the privilege of publishing two review articles on cancer biology from Sophia Tsai and Ming-Jer Tsai [1], and Hui-Kuan Lin [2].

Drs. Sophia and Ming-Jer Tsai are prominent scientists in the field of gene regulation and gene function. Dr. Sophia Tsai holds the Gordon Cain endowed professorship in the Department of Molecular and Cellular Biology and the Department of Medicine, Baylor College of Medicine, Houston, Texas. Professor Tsai received her Bachelor and Master degrees in Chemistry from the University of Wisconsin, Madison, Wisconsin, and her PhD degree in Biochemistry from the University of California, Davis. Following her postdoctoral training at Cornell University, Ithaca and M.D. Anderson Cancer Center, Houston, she joined the Cell Biology Department at Baylor College of Medicine, Houston, TX and remained there since. Dr. Ming-Jer Tsai is Charles C. Bell Distinguished Service Professor in the Department of Molecular and Cellular Biology, Baylor College of Medicine, Houston, Texas. Professor Tsai received his Bachelor in Science from National Taiwan University and PhD in Biochemistry from the University of California, Davis. After his postdoctoral training in the University of Texas, M.D. Anderson Cancer Center, he was recruited to the Department of Molecular and Cellular Biology, Baylor College of Medicine, as a faculty member in 1973 and remained there since. Professor Tsai is an Academician of Academia Sinica, Taiwan.

Drs. Sophia and Ming-Jer Tsai have played a pivotal role in creating a new field on nuclear orphan receptors. When they and their colleagues first cloned COUP-TFI in 1989, they were among the first researchers to clone novel factors that were related to steroid receptors but not have known ligands or physiological function. Subsequently, they cloned another highly related family member, COUP-TFII. These factors became known as orphan receptors which over the years have expanded the steroid receptor family to a superfamily of 48 members in humans. They showed that COUP-TFI and II are essential for cell fate determination during embryonic development and established them as early critical players in normal development, controlling cell-fate specification, neural development, organogenesis, angiogenesis and metabolism. Dr. Tsai’s team also showed that dysregulation of COUP-TFII is the major underlying reason for serious diseases, such as congenital diaphragmatic hernia, congenital heart defects, tumorigenesis and heart failure. Their review article is focusing on the roles of COUP-TFII in tumorigenesis and tumor metastasis.

Dr. Hui-Kuan Lin is currently holding an Associate Professor position and R. Lee Clark scholar in the Department of Molecular and Cellular Oncology, The University of Texas MD Anderson Cancer Center, Houston, Texas. Dr. Lin received his Bachelor’s and Master’s degrees in Pharmacy and Pharmacology from National Taiwan University, Taipei, Taiwan 1993 and 1995, respectively. He then moved to the University of Rochester, Rochester, New York for his Ph.D study of Cancer Biology until his graduation in 2002. Afterwards, Dr. Lin went to the Memorial Sloan-Kettering Cancer Center, New York, for his post doctoral training in Cancer Biology and Genetics with Dr. Pier Paolo Pandolfi. He was recruited as a faculty member by the University of Texas Health Science Center and MD Anderson Cancer Center, Houston, Texas in 2007 and promoted to Associate Professor in 2011.

Dr. Lin is an outstanding junior investigator with his research focusing on the roles of posttranslational modifications of proteins in cancer signaling. Dr. Lin made important contributions to the understanding of key signal pathways, such as PI3K/PTEN/Akt and TGF-beta, involved in regulation of cancer development. His recent work has been directed to understand the role of posttranslational regulation of protein factors in cancer development. In the review article, Dr. Lin described Akt activation through posttranslational modifications, including phosphorylation, OGlcNAcylation, ubiquitination, SUMOylation and acetylation.

SCBA appreciates scientific contributions by its outstanding members like Drs. Sophia and Ming-Jer Tsai, and Hui-Kuan Lin since these contributions will eventually benefit all human beings for better health and life.
